# Appearance of Hysteresis Phenomena on Hydrodynamic Lubrication in a Seal-Type Thrust Bearing with Dimples

**DOI:** 10.3390/ma14185222

**Published:** 2021-09-10

**Authors:** Reo Miwa, Norifumi Miyanaga, Jun Tomioka

**Affiliations:** 1 Graduate School of Engineering, Kanto Gakuin University, 1-50-1 Mutsuurahigashi, Yokohama 236-8501, Japan; d21j8002@kanto-gakuin.ac.jp; 2 Department of Science and Engineering, Kanto Gakuin University, 1-50-1 Mutsuurahigashi, Yokohama 236-8501, Japan; 3 Faculty of Science and Engineering, Waseda University, 3-4-1 Okubo, Shinjuku-ku, Tokyo 169-8555, Japan; tomioka@waseda.jp

**Keywords:** hydrodynamic lubrication, thrust bearings, hysteresis phenomena, dimples

## Abstract

This paper described unique hysteresis phenomena that appear in the hydrodynamic lubrication properties of dimpled thrust bearings. A seal-type thrust bearing specimen was textured with dimples. The load-carrying capacity and frictional torque were measured with a constant film thickness and compared to those of a dimple-free specimen. For examining the size of cavitation bubbles that occurred in various conditions, the lubricating area was observed during experiments. The used dimpled specimen produced the load-carrying capacity, and it exhibited an interesting hysteresis phenomenon, the difference in the values in the increasing and decreasing processes of rotational speed. The visualization test results revealed that the size of cavitation bubbles occurring within the dimples strongly affected this phenomenon. In addition, the dimpled specimen was able to reduce the frictional torque compared to the dimple-free specimen. However, the frictional torque did not show the hysteresis loop similar to that shown in the load-carrying capacity.

## 1. Introduction

Applying dimples and/or grooves to the lubricating area is an effective method for improving lubrication performance [1]. Consequently, this technology is being used in various mechanical elements such as bearings [2,3,4,5], seals [6,7,8], and piston rings [9,10].

In 1996, Etsion et al. [11] calculated the frictional torque and leakage in a mechanical seal with a regular microsurface structure. They concluded that an increase in the load-carrying capacity (fluid film force) was obtained owing to cavitation in each dimple. This is an acceptable mechanism to explain the enhancement of lubrication performance on dimpled surfaces. Dimples contain diverging and conversing areas; hence, a fluid film cavitates within the diverting area. Owing to the emerged cavitation, the fluid pressure in the area cannot drop below the cavitation pressure at which cavitation starts to occur, and thus, it generates a load-carrying capacity for supporting applied loads. Other promising mechanisms for the expansion of hydrodynamic lubrication have also been proposed [12,13,14,15].

These publications have motivated subsequent researchers to investigate this topic, and the effects of surface textures have been actively studied recently with the objective of finding an optimal selection of the dimple size and density. For this purpose, experimentally understanding the behavior of the fluid film through the measurements of load-carrying capacity and friction and visualizations of the contact area. Shen et al. [16] compared rectangular and triangular cross-sectional profiles and concluded that a rectangular profile could produce a higher load-carrying capacity. Yu et al. [17] calculated the hydrodynamic pressures of circular, triangular, and elliptical dimples. Cross et al. [18] performed visualization tests of circular pocket thrust bearings and reported that the cavitation area increased with the increase in rotational speed and lubricant viscosity. However, the effects of cavitation on the hydrodynamic lubrication of a dimpled surface have not been fully clarified. One of the problems for clarifying the cavitation effects is that for balancing with applied load, the fluid film thickness changes in conventional test devices. Therefore, the discussion of the cavitation effects on the bearing performance has included the film thickness effects.

To overcome this difficulty, we [19] have developed an experimental device wherein the film thickness can be kept constant, and the load-carrying capacity and frictional torque are measured under various conditions. Using the device, the effects of the dimple shape on the hydrodynamic properties are discussed. This device allows discussing the cavitation effect with changing sliding speed and provides additional insight into the hydrodynamic characteristics of dimpled thrust bearings. The previous paper implied that the size of cavitation bubbles that appeared in the dimples had a considerable impact on the load-carrying capacity and frictional torque.

In this paper, we showed the hysteresis phenomena that appear in the hydrodynamic lubrication properties of a seal-type thrust bearing. The load-carrying capacity and frictional torque were measured with a fixed film thickness condition. For examining the size of the cavitation bubbles that occurred in various conditions, the lubricating area was observed during experiments. Based on these results, the relationship of the occurrence of cavitation to the hysteresis phenomena of the load-carrying capacity and frictional torque was discussed. 

## 2. Experimental Apparatus and Methods

### 2.1. Experimental Device

**Figure 1:**
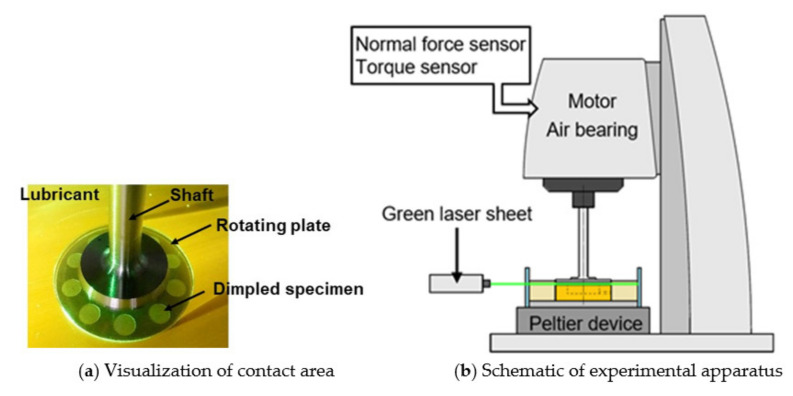
Experimental apparatus; contact area is lighted by a green laser for viewability.

Figure 1 shows the experimental apparatus used herein. This device allows controlling the rotational speed, the gap (fluid film thickness) between upper and lower plates, and the bulk temperature of the lubricant. A parallel thrust bearing comprises an upper glass plate (*Ra* = 0.009 µm) and a lower brass specimen (*Ra* = 0.040 µm). The glass plate rotates with a shaft supported by an aerostatic bearing. Through the glass plate, it is possible to observe cavitation occurring in the dimples. The lubricating surface is irradiated by a seat-type green laser for the visibility of cavitation bubbles. A digital camera is installed adjacent to the experimental setup. It is focused on the stationary specimen and can view seven or eight dimples at the same time.

The specimen is fixed on a Peltier device. In this apparatus, the gap between the glass plate and the specimen can be controlled with an accuracy of 1 μm. The load-carrying capacity and frictional torque acting on the glass plate by the fluid film can be measured using a normal force sensor and torque sensor, respectively. The lubricant is impounded in an oil cup, and the bulk temperature of the lubricant was controlled by the Peltier device.

### 2.2. Specimens

**Figure 2:**
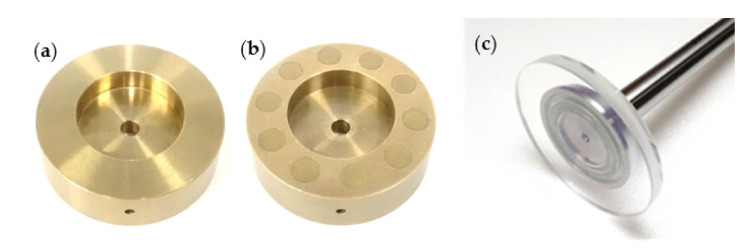
Photos of the specimens: (**a**) plane (dimple-free) specimen, (**b**) dimpled specimen, and (**c**) glass plate.

**Figure 3:**
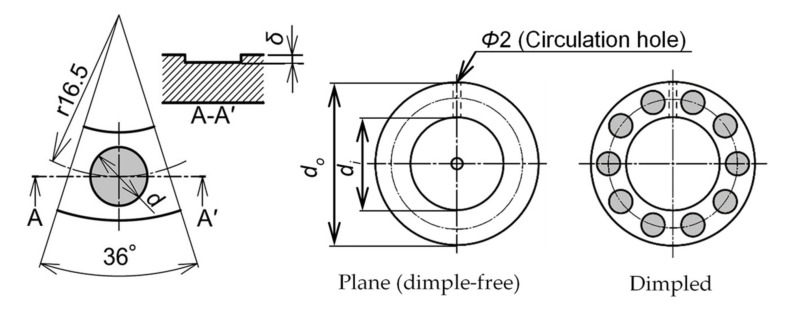
Details of the specimen used in this study (*d* = 6 mm, *δ* = 30 μm, *d_i_* = 24 mm, and *d_o_* = 42 mm).

Figure 2 shows images of the specimens used in this study, while Figure 3 illustrates the details of the specimens used. The inner and outer diameters of the specimens were 24 and 42 mm, respectively. For the dimpled specimen, the circular dimples were manufactured on the lubricating surface by chemical etching. The details of the etching process are described in the literature [20]. The depth of dimples can be controlled by the etching time.

**Figure 4:**
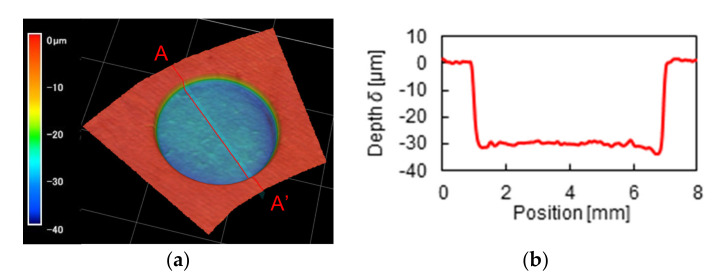
Microscope image of dimples used in this study: (**a**) 3D profile of a dimple, (**b**) Cross-sectional profile on A-A′.

In this study, ten dimples with a diameter of 6 mm and a depth of 30 μm were evenly located at the medium radius of the specimen. Figure 4 shows a microscope image of the dimple. Each dimple had an almost square-shaped bottom. The dimple area ratio (the ratio of dimple area to lubricating area) was 30% in this study. The plane (dimple-free) specimen was also tested for comparison, whose inner and outer diameters are the same as the dimple specimen.

In the side wall of dimple and plane specimens, for lubricant replacement, a 2 mm-diameter circulation hole was constructed. The holes ensure that the pressures of the inner and outer sides of the lubricating area are maintained at the ambient pressure.

### 2.3. Experimental Procedure

In this device, the gap between the upper glass disk and the lower specimen can be kept constant with an accuracy of 1 μm, and with the fixed film thickness, the load-carrying capacity can be recorded. The frictional torque acted between two surfaces was measured at the same time. At each rotational speed, pictures of the lubricating area were taken.

The test procedure involved the same sequence for the dimple and plane specimens. To begin the test, the lower specimen was placed on the Peltier device and submerged in lubrication oil. The shaft with the glass plate was attached to a spindle, and the film thickness was fixed at a constant value of 30 μm, basing on the consideration of that almost same film thickness with groove/dimple depth show better bearing performance [5,16].

**Figure 5:**
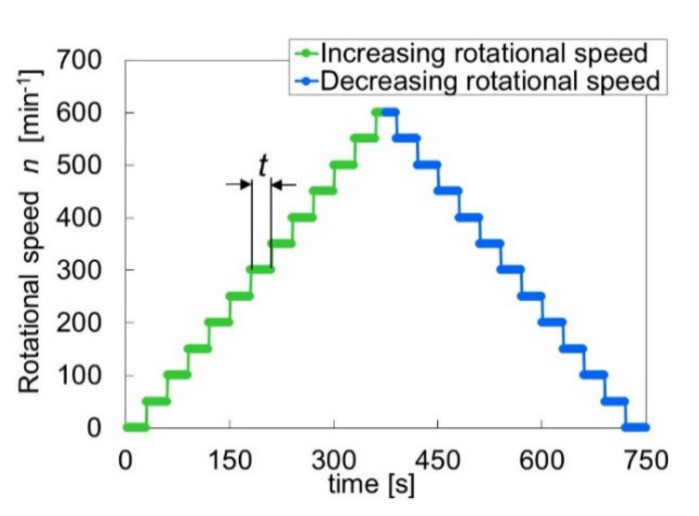
Control of the rotational speed.

**Figure 6:**
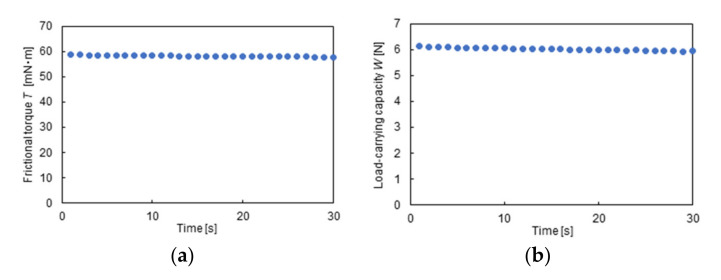
An example of measurement results, *n* = 400 min^−1^, *h* = 30 μm: (**a**) load-carrying capacity; (**b**) frictional torque.

The rotational speed was increased and then decreased in a stepwise manner. Figure 5 shows the history of rotational speed. The rotational speed was increased up to 600 min^−1^ with an increment of 50 min^−1^ and then decreased to 0 min^−1^ with a decrement of 50 min^−1^. The test period for each rotational speed was 30 s, and the average data were recorded. Figure 6 shows an example of measurement results for the load-carrying capacity and frictional torque.

For examining the cavitation occurring in the dimples and its effects on the load-carrying capacity and frictional torque, the lubricating area was observed during the measurements. Cavitation occurring in the dimples was captured by a digital camera. The area ratio to the dimple was analyzed for each experimental condition.

The bulk temperature of lubricant was maintained at 298 ± 0.1 K using the Peltier device. SAE30 oil was used in the experiments, which has a viscosity of 0.22 Pa·s at 298 K. The experimental conditions used herein are listed in Table 1.

## 3. Experimental Results and Discussion

**Figure 7:**
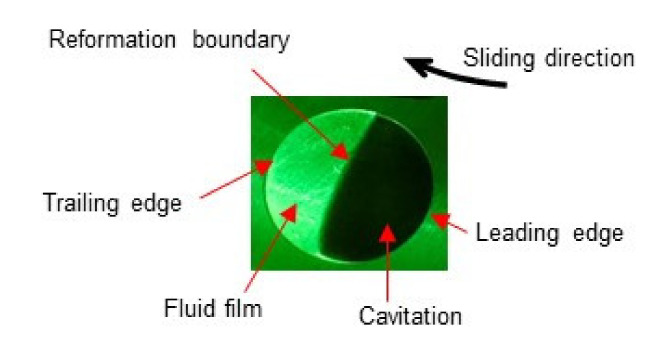
Example of cavitation occurred within a dimple.

Figure 7 shows an example of cavitation occurring within a dimple. The upper glass plate was moved from the right to the left. The contact area was illuminated by a green laser. A cavitation bubble appeared at the leading edge of the dimple and conformed to a circular shape. The reformation boundary between the cavitation area and the fluid film area was clearly determined within the dimple. The reformation boundary is slightly convex to the flow direction.

It is known [21] that sliding a flat surface against a curved surface separated with lubricant film results in the generation of positive hydrodynamic pressure in the convergent section of the gap and negative pressure in the divergent section. The dimple used in this study provides the convergent-divergent gap just described. Thus, it is expected that negative pressure occurs at the leading edge. When the negative pressure is lower than the cavitation pressure (gaseous pressure or vapor pressure) at which cavitation starts to occur, cavitation babbles appear in the dimple. The result shown in Figure 7 clearly responded to the expectation.

**Figure 8:**
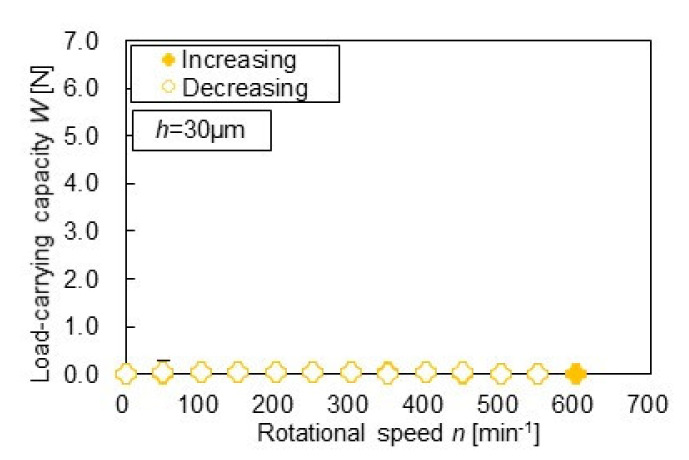
The load-carrying capacity for plane specimen.

**Figure 9:**
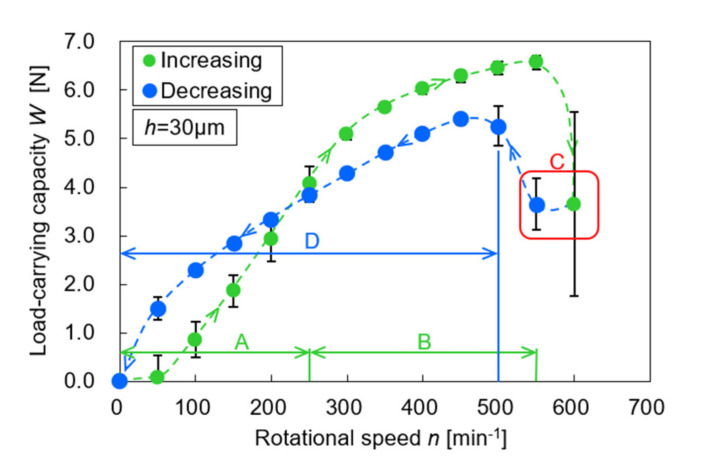
The load-carrying capacity of dimple specimen.

**Figure 10:**
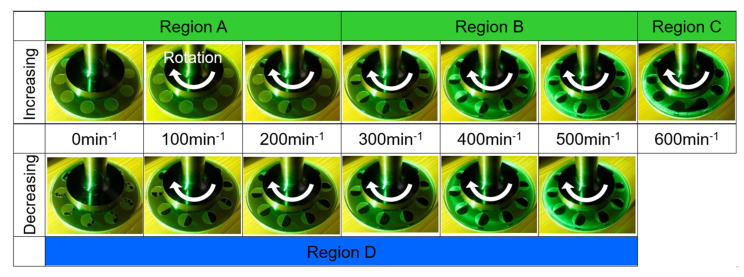
Observation results of the lubricating surface.

Figure 8 and Figure 9 show the relation between the measured load-carrying capacity and rotational speed. Plot means the averaged value, whereas the error bar means the standard deviation. The variation in the measurement results was very small. As shown in Figure 8, the plane specimen did not generate the load-carrying capacity in both the increasing and decreasing processes because it did not have the convergent portion of the gap. This result has a good agreement with the fact that the parallel flat bearings cannot produce the hydrodynamic pressure and its integration value, i.e., the load-carrying capacity [21]. On the contrary, as shown in Figure 9, the dimpled specimen generated the load-carrying capacity, and interestingly the increasing and decreasing process of the rotational speed showed the different values. Consequently, the hysteresis phenomenon that had two loops appeared. No paper has been described and discussed this phenomenon on dimpled thrust bearings ever, hence the present result provides additional insight into the hydrodynamic characteristics of dimpled thrust bearings. With the visualization results shown in Figure 10, the results are discussed below.

In region A of Figure 9, cavitation bubbles appeared in some dimples. For example, at 200 min^−1^, only three dimples had a cavitation bubble, as shown in Figure 10; thus, the other dimples did not generate the load-carrying capacity. In addition, the number of film-ruptured dimples increased with the rotational speed. This means that in this region, the increase in the load-carrying capacity was affected by not only the rotational speed but also the occurrence of cavitation.

When the rotational speed reached region B, from 300 to 550 min^−1^, all dimples had a cavitation bubble. The cavitation bubble expanded with rotational speed. On the contrary, the load-carrying capacity also increased with the rotational speed, but that in region B was more gradual than that in region A. It is considered that in this region, as the cavitation bubbles already appeared in all dimples, the increment of the load-carrying capacity was affected mainly by the rotational speed.

In region C, the cavitation bubbles in each dimple overflowed, sometimes connecting with those in the next dimples, and grew similar to one large ring. In this region, the load-carrying capacity rapidly decreased, and the value was unstable and hence showed a larger standard deviation. This is because that the dimples are covered with cavitation bubbles, thereby preventing the hydrodynamic effect expected for the dimples. In addition, it should be considered that in the bearing designs, this unstably generated load-carrying capacity results in a self-excited vibration of the bearings.

**Figure 11:**
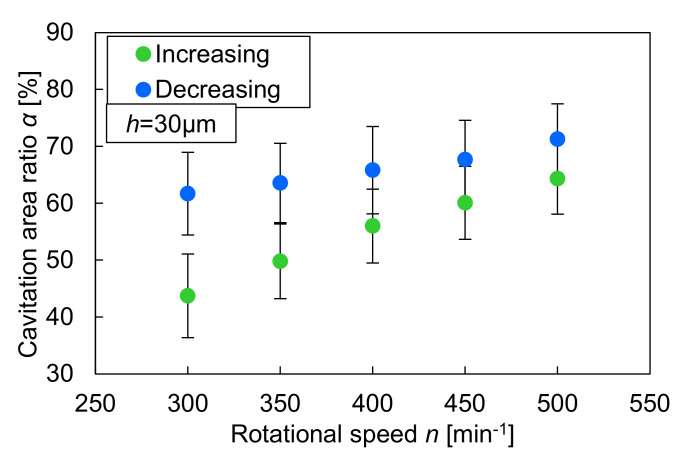
The relationship between cavitation area ratio and rotational speed.

Subsequently, with a decrease in the rotational speed, the cavitation bubbles backed to their dimples, and the load-carrying capacity increased and stabilized. In region D, the load-carrying capacity gradually decreased as the rotational speed decreased. In addition, it was smaller than that of the increasing process, even at the same rotational speed. When comparing the visualization results at 300, 400, and 500 min^−1^, in which all dimples had cavitation bubbles in both processes, the cavitation bubbles of the decreasing process were observed to be larger than those of the increasing process. Figure 11 shows the relation between the cavitation area ratio and the rotational speed. The cavitation area ratio is the ratio of the cavitation area to the dimple area, as shown in Equation (1).

(1) $$\alpha =\frac{A_{cav}}{A_{d}}$$

where α is the cavitation area ratio, *A_cav_* is the area of cavitation, and *A_d_* is the area of the dimple. The ratio was obtained from the average of 10 dimples in the three experiments. The variation in the measurement results of the cavitation area ratio is relatively large, but the difference between the increasing and decreasing processes is obvious. It can be said that the size of the cavitation bubbles affected the load-carrying capacity. In contrast, at a rotational speed of approximately 200 min^−1^, the results in the increasing and decreasing processes crossed. In the decreasing process, region D, 10 dimples had a cavitation bubble, and the load-carrying capacity was greater than that of the increasing process in region A, wherein a few dimples generated the load-carrying capacity. As shown in Figure 9 and Figure 10, the cavitation bubbles shrink as the rotational speed decreases. When the glass plate stopped rotating, the cavitation bubbles split immediately and stayed within the dimples.

In the dimpled thrust bearing used herein, it is expected to occur either gaseous cavitation or vapor cavitation. When the hydrodynamic pressure decreases below the gaseous pressure, dissolved gases are released in the form of bubbles. If the pressure further decreases to below the vapor pressure, the lubricant evaporates. For the vapor cavitation, bubbles are expected to collapse and change back to liquid form immediately after the glass plate stops rotating. The present result indicated that the cavitation bubbles were gaseous as the cavitation bubbles remained within dimples. Since the air solubility in lubricant is very small [22], it is expected that the dissolved gases are not able to immediately solve into the lubricant. It is considered, therefore, that larger quantities of gas released from the lubricant at a higher rotational speed in the increasing process had to remain even in the decreasing process. Consequently, the decreasing process had a larger cavitation area ratio and lower load-carrying capacity than the increasing process. It can be concluded that this is the reason for the appearance of the hysteresis phenomena on the load-carrying capacity.

**Figure 12:**
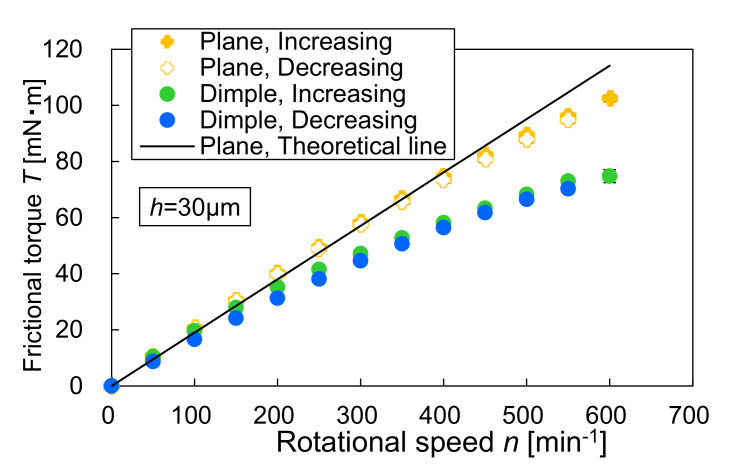
The relationship between frictional torque and rotational speed.

Figure 12 shows the relation between frictional torque and rotational speed. The figure includes the theoretical result for the plane specimen. The theoretical frictional torque is calculated using the following equation [21].

(2) $$T=\frac{\pi \eta \omega }{32h}\left(d_{0}^{4}-d_{i}^{4}\right)$$

where *T* is the frictional torque, *h* is the film thickness, *η* is the viscosity of the lubricant, and *ω* is the angular velocity of the rotating glass plate. As shown in Figure 12, the frictional torque of the plane specimen was in good agreement with the theory. The dimpled specimen exhibited a lower frictional torque than the plane specimen. This trend was more remarkable at higher rotational speeds. For the dimpled specimen, the frictional torque during the decreasing process was slightly lower than that during the increasing process. This is because in the decreasing process, the cavitation bubbles were larger, and the shear stress decreased. However, these values were very close, and the hysteresis loop like that in the load-carrying capacity was not observed. It is can be concluded that for the dimpled thrust bearings, the rotational speed is dominant in the frictional torque subjected to a constant gap than the cavitation bubbles that occur in the dimples.

## 4. Conclusions

In this study, we discussed the hysteresis phenomena of the hydrodynamic lubrication properties of the seal-type thrust bearings. The load-carrying capacity and frictional torque were measured with a fixed film thickness for separating the effect of changing film thickness. The visualization results of the lubricating area during measurements were used for the discussion of the hysteresis phenomena.

The dimpled specimen produced the load-carrying capacity, but the value of the decreasing process of the rotational speed was lower than that of the increasing process. The reason related to the result was the size of the cavitation bubble. In the decreasing process, larger sized cavitation bubbles were observed. In the higher rotational speed (the region C), the cavitation bubbles overflowed and sometimes connected with those in the next dimples. When this connection occurred, the load-carrying capacity rapidly decreased, and the value was unstable. It should be considered that in the bearing designs, this unstably generated load-carrying capacity results in a self-excited vibration of the bearings. The values of the frictional torque in the increasing and decreasing processes were very close, and the hysteresis phenomena in the frictional torque were not clear.

The numerical research on the hysteresis phenomena is necessary for further understanding. It would be important to obtain the relation of the cavitation pressure and air solubility in various operating conditions. The dimple parameters such as depth, diameter, area ratio and materials of lubricants and specimens interact in a complex manner. Further research on their optimization is necessary to improve the bearing performance.

## Figures and Tables

**Table 1:** Experimental conditions.

Rotational speed, *n*, (min^−1^)	0–600
Test period of each condition, *t*, (s)	30
Film thickness, *h*, (μm)	30
Bulk temperature of lubricant, *T*, (K)	298 ± 0.1
Viscosity of lubricant, *η*, (Pa·s)	0.22
Lubricant	SAE30

## Data Availability

The data presented in this study are available on request from the corresponding author after obtaining permission of authorized person.
